# Biological and Pharmacological Aspects of the NK1-Receptor

**DOI:** 10.1155/2015/495704

**Published:** 2015-09-03

**Authors:** Susana Garcia-Recio, Pedro Gascón

**Affiliations:** ^1^Laboratori d'Oncologia Molecular i Traslacional, Fundació Clínic per a la Recerca Biomèdica, 08036 Barcelona, Spain; ^2^Departament de Medicina, Universitat de Barcelona, 08036 Barcelona, Spain

## Abstract

The neurokinin 1 receptor (NK-1R) is the main receptor for the tachykinin family of peptides. Substance P (SP) is the major mammalian ligand and the one with the highest affinity. SP is associated with multiple processes: hematopoiesis, wound healing, microvasculature permeability, neurogenic inflammation, leukocyte trafficking, and cell survival. It is also considered a mitogen, and it has been associated with tumorigenesis and metastasis. Tachykinins and their receptors are widely expressed in various human systems such as the nervous, cardiovascular, genitourinary, and immune system. Particularly, NK-1R is found in the nervous system and in peripheral tissues and are involved in cellular responses such as pain transmission, endocrine and paracrine secretion, vasodilation, and modulation of cell proliferation. It also acts as a neuromodulator contributing to brain homeostasis and to sensory neuronal transmission associated with depression, stress, anxiety, and emesis. NK-1R and SP are present in brain regions involved in the vomiting reflex (the nucleus tractus solitarius and the area postrema). This anatomical localization has led to the successful clinical development of antagonists against NK-1R in the treatment of chemotherapy-induced nausea and vomiting (CINV). The first of these antagonists, aprepitant (oral administration) and fosaprepitant (intravenous administration), are prescribed for high and moderate emesis.

## 1. Tachykinins and Their Receptors

The tachykinins are one of the largest conserved families of peptides involved in neurotransmission and inflammatory processes. The idea that tachykinins act exclusively as neuropeptides is currently being challenged. Substance P (SP), a small undecapeptide present in both mammalian and nonmammalian species, was the first member of the family to be discovered (as early as 1931, by von Euler and Gaddum). SP is associated with multiple processes: hematopoiesis, wound healing, microvasculature permeability, neurogenic inflammation, leukocyte trafficking, cell survival, and metastatic dissemination [1–5]. The three classical members of the mammalian tachykinin family are SP and neurokinin A (NKA), both encoded by the TAC1 gene, and neurokinin B (NKB), encoded by the TAC3 gene. A third mammalian tachykinin gene (TAC4) codes for hemokinins and endokinins [1, 6, 7]. The* TAC1* gene (according to the Human Genome Organization (HUGO) Gene Nomenclature Committee (http://www.genenames.org/) also encodes other tachykinins, including NKA, neuropeptide K (NPK). and neuropeptide *γ* (NP*γ*). On the other hand, the* TAC3* gene only codes for NKB (previously known as* PPT-B* gene). In 2000, Zhang et al. identified a third gene called* TAC4* (previously named* preprotachykinin-C* (*PPT-C*)) and demonstrated its association with the hematopoietic system and the maturation of B lymphocytes [7]. This gene encodes hemokinin 1 (HK-1) and its shorter derivative hemokinin (4–11) and four other peptides called endokinins (EKS), EKA, EKB, EKC, and EKD [6].

Tachykinin receptors have been divided into three different types according to their affinity ligands (high or low): TACR1 (NK-1 receptor), TACR2 (NK-2 receptor), and TACR3 (NK-3 receptor) (Table 1), which have preferential (but not exclusive) affinities for SP, NKA, and NKB respectively [8–10]. The order of potency of these receptors per tachykinin is shown as follows [10, 11]. Order of affinity of tachykinin receptor by its agonists is

- Receptor NK-1: SP>NKA>NKB;
- Receptor NK-2: NKA>NKB>SP;
- Receptor NK-3: NKB>NKA>SP.

NP*γ* and NPK preferentially bind to the NK-2 receptor. The affinities of NKA and NKB for the NK-1 receptor are, respectively, 100 and 500 times lower than that of SP [12]. It has also been reported that SP interacts with fibronectin (FN) and hematopoietic growth factor inducible neurokinin-1 type (HGFIN) [13, 14]. The homology between the NK1 receptor and HGFIN has recently been described. This finding may be relevant because both the NK-1 receptor and HGFIN have been linked to tumorigenesis, including breast cancer (BC) [14]. However, whereas the NK-1 receptor has been described as a tumor promoter, HGFIN may act as a suppressor [14].

**Figure 1:**
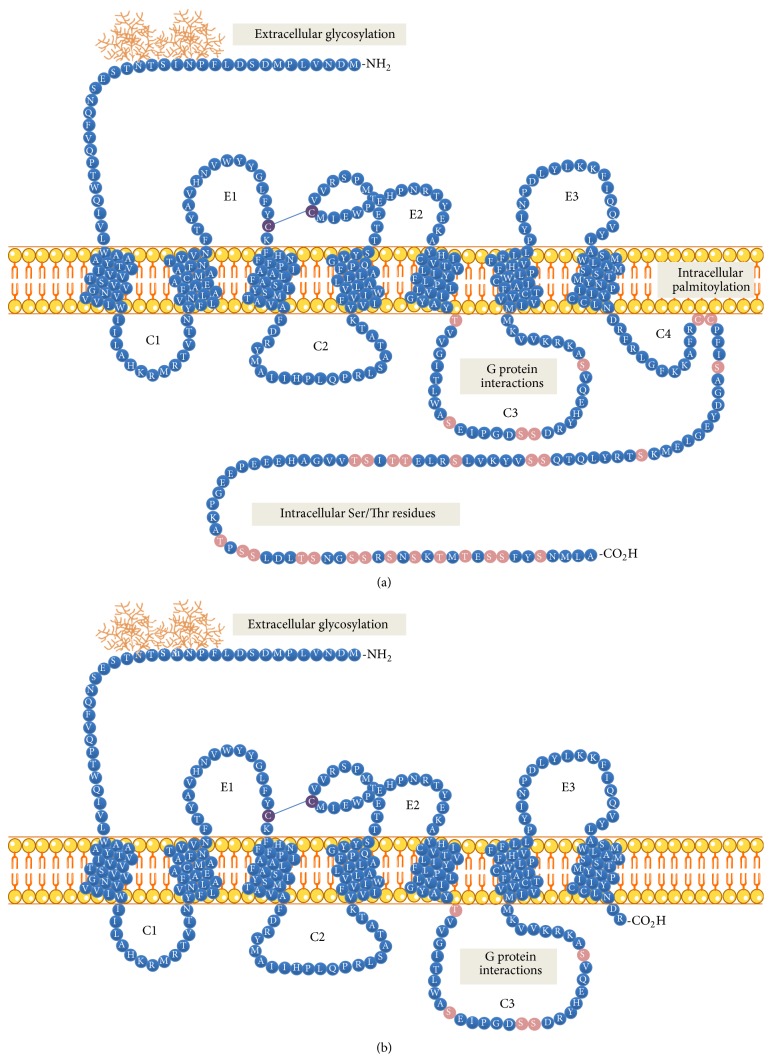
Schematic model of the NK-1 receptor. (a) Complete isoform or long isoform-full length (NK1-FL) with 407 amino acids. It contains an extracellular N-terminus, seven transmembrane domains, three extracellular loops (E1, E2, and E3) and three intracellular loops (C1, C2, and C3), a possible C4 because of a Cys palmitoylation residue and an intracellular C-terminus. Asn14 and Asn18 are given as putative glycosylation sites. (b) Depiction of the truncated isoform with 311 amino acids, showing that this isoform has lost a part of the C-terminal end, and also the intracellular Ser/Thr residues responsible for internalization. Modified from [149].

The three tachykinin receptors belong to family 1 (rhodopsin-like) G protein-coupled receptors (GPCRs) and are encoded by five exons [9, 15]. These are seven-transmembrane-helix receptors which share the same structural unit: three extracellular (EL1, EL2, and EL3) and three intracellular loops (C1, C2, and C3) with the possibility of a fourth loop, due to the palmitoylation of cysteine (Cys), flanked by seven intermembrane domains (TM 1-VII), and an amino-terminal extracellular and carboxy-terminal cytoplasmic domain [9] (Figure 1).

The carboxy-terminal conserved domain of tachykinins (Phe-X-Gly-Leu-Met-NH2) interacts with tachykinin receptors, while the amino-terminal sequence is responsible for the specificity of the receptor [16]. All tachykinins are amidated at the C-terminal and deamidation suppresses their activity [8]. The second and third loops are involved in the binding of agonists or antagonists, while the third cytoplasmic loop is responsible for binding to protein G. The C-terminus contains serine/threonine residues which, once phosphorylated, cause desensitization of the receptor when it is repeatedly activated by the agonist. The 5′ region of the gene has several putative regulatory DNA elements such as the cAMP responsive element, AP-1, AP-2, AP4, NF-кB, OCT-2, and a domain Sp-1 [16]. Specifically, the NK-1 receptor has 407 amino acids and a relative molecular mass of 46 kDa [17]. NK-2 and NK-3 consist of 398 and 465 amino acids, respectively, NK-3 being the longest of the three receptors. The most important splicing identified loses the last 96 amino acids at the C-terminus and thus has 311 amino acids [18–20] (Figure 1). This shorter or truncated isoform (NK1-Tr) is generated when the intron located between exons 4 and 5 is not removed and the premature stop codon is identified before starting exon 5.

Lai et al. [21] observed that SP specifically increased intracellular calcium in embryonic kidney cells (HEK293) stably transfected with the long isoform, while there was no effect in those transfected with the truncated isoform. Likewise, cells expressing the long isoform activated NF-B and IL-8, while those expressing the truncated one had a lower mRNA expression of IL-8 and were unable to activate NF-кB. The activation of protein kinase Erk was also altered in the same cells: whereas phosphorylation of this protein through the long isoform was fast (1 to 2 minutes) and sustained, cells transfected with truncated isoform were not able to phosphorylate Erk protein within 20 min after exposure to SP [21]. In addition, other studies have demonstrated that SP had a lower relative affinity for the truncated receptor form (up to 10 times less than the full isoform) [18]. Moreover, the loss of certain C-terminal serine and threonine residues is important for G protein-coupled receptor kinase (GRK) interaction and *β*-arrestin recruitment for subsequent receptor internalization [22–24].

Therefore, the truncated form should be capable of prolonging the responses after ligand binding because its desensitization and internalization are affected. Besides the differences between the two isoforms, another important phenomenon involved in the receptor signaling should be mentioned. Tansky, Leeman, and Pothoulakis showed that the amino terminal end had two glycosylated Asn (N-) sites and described how these glycosylations can influence the functional level of the receptors [25].

They observed that nonglycosylated receptors showed half the affinity for SP shown by glycosylated receptors, and in fact the nonglycosylated NK-1 receptor was internalized faster than the glycosylated form. This also suggested the possibility that glycosylation may be a feature in the stabilization of the receptor in the plasma membrane. Several bands of different molecular weights have been identified, probably due to this phenomenon. For example, in lymphocytes, certain forms of glycosylated receptor (58 kDa) have been described [26], while others with bands of 38 and 33 kDa appear in IM-9 lymphoblasts (26). Furthermore, isoforms with bands of 75, 58, 46, and 34 kDa have been identified in several studies of tumor pancreatic carcinoma cell lines [27, 28].

In the past two decades, other isoforms have been identified besides the conventional ones, with different SP affinities. For example, in rat salivary glands another apparently truncated isoform has also been detected in the C-terminal end, with 8 kDa less than the long isoform [29]. Li et al. also demonstrated that the short isoform seems to have an SP affinity similar to that of the complete isoform. It has been suggested that this isoform comes from posttranslational modifications [30]. In addition, other studies have shown that some receptor isoforms present different affinities from the “classic” forms. This has led to a division of the NK-1 receptor into three different classes: (1) the “classic” NK-1 receptor (which shows greater binding affinity for the SP ligand), (2) the “sensitive to septide” NK-1 receptor (showing a very similar affinity for binding to SP and other tachykinins as NKA, NPK, NP*γ*, NKB, and even other synthetic peptides such as septide fragment 6–11 SP, which gives the receptor its name) [10, 31], and (3) the “new NK-1 sensitive” receptor [32]. This subtype has a higher affinity for longer tachykinins and does not bind to septide or SP (6–11). However, more studies are needed to identify the real differences in the signaling pathways of each NK-1R isoform and the preferred sites of expression of the different isoforms or glycosylated forms.

### 1.1. Signaling Pathways Modulated by Tachykinins and Their NK-1R

The physiological processes mediated by SP or other tachykinins occur via the NK-1 receptor, which belongs to the large family of G-protein-coupled receptors (GPCRs). Via second messengers, G proteins activate transduction pathways within the cell. Which pathways are activated by G proteins depends on the nature of the proteins belonging to this large family: for example, the activation of NF-*κ*B mediated by SP, interleukins, or growth factors (IL-1, IL-6, IL-8, TNF-*α*, and IFNy) and the activation of MAPKs pathway or PI3K/Akt among others [33–35].

#### 1.1.1. GPCR-Mediated Signal Transduction: Classification and Function of G Proteins

GPCRs mediate their signaling through heterotrimeric G proteins transmitting signals from a variety of surface cell receptors to enzymes and ion channels. This complex is composed by three distinct subunits: the G*α* subunit that binds to GDP/GTP and the G*β* and G*γ* subunits that form the G*βγ* complex (which present strong bindings between them) [36, 37]. After binding SP to the specific NK-1 receptor, a change occurs in the G*α* subunit, allowing it to exchange GTP for GDP and permitting the dissociation of the G*βγ* dimer. These subunits (G*α* and G*βγ*) begin their own signaling cascade separately and positively or negatively regulate the activity of enzyme effectors and ion channels that are cell type- or GPCR-specific [38, 39].

**Figure 2:**
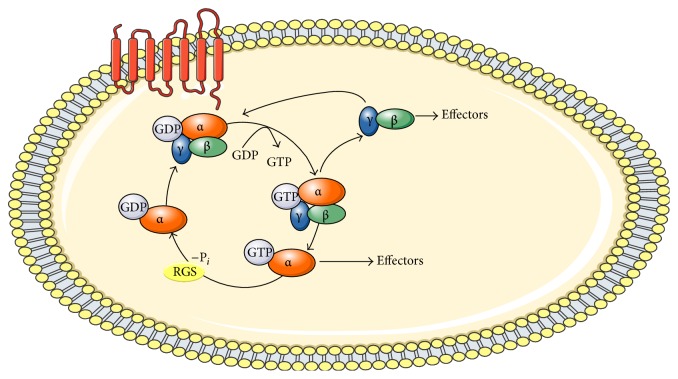
Heterotrimeric G protein activation by GTP and consequent separation of subunits. Heterotrimeric G proteins have been grouped into four distinct families based on the G_*α*_ amino acid homologous sequence: G_s_, G_i_, G_q_, and G_12/13_. There are two major signaling pathways associated with G_*α*s_ and G_*α*q_ subunits and are mainly how NK-1 receptor signals [48, 50, 59]. The different signaling pathways activated by each subunit will be explained below. This figure was made using Servier Medical Art collection (http://creativecommons.org/licenses/by/3.0).

The GTP hydrolysis returns the G*α* subunit to its inactive state, allowing again the trimeric formation with the G*βγ* subunit [40]. G*βγ* in contrast to the G*βγ* subunit, the broad range of the *α* subunit is limited because all *α* subunits, except G_*α*t_, have a palmitic acid posttranslational modification in the amino-terminal portion, which keeps them adhered to the plasma membrane [41]. The *α* subunit itself has intrinsic GTPase capacity and may modulate its own inactivation. In any case, this GTP hydrolysis is relatively low compared with other accessory proteins called cytoplasmic regulators of G protein signaling (RGS) [42] (Figure 2).

(i) G_q/11_: the receptor interaction by the agonists regulates the activation of G_q/11_ protein and the subsequent activation of phospholipase C*β* (PLC*β*), which degrades the phosphatidylinositol 4, 5-bisphosphate (PIP2) to produce two compounds: diacylglycerol (DAG) and inositol 1,4,5-triphosphate (IP3), responsible for increasing intracellular calcium [43–47].
(ii) G_s_: this subunit is responsible for the activation of the second messenger adenylate cyclase (AC), which catalyzes the conversion of cytoplasmic ATP into cyclic adenosine monophosphate (cAMP) when the Gs-related pathway is activated (by contrast, AC inhibition is conducted by the Pertussis toxin-sensitive G_i_-protein (PTX) in rat submandibular cells) [48]. Other studies have reported that the Gs subunit is the substrate of cholera toxin (CTX), produced by* Vibrio cholerae*, which catalyzes its ADP ribosylation and inhibits its intrinsic GTPase activity [42]. It has been widely reported that increased cAMP levels lead to activation of protein kinase A (PKA). Activation of PKA, then, phosphorylates the transcription factor CREB (cAMP-responsive element-binding protein CRE). CREB binds to the cAMP response element (CRE) of a target gene and negatively affects the activation of NF-кB [49]. However, despite the Gs action, the power to generate cAMP accumulation by NK-1R agonists is lower than the ability to induce IP_3_ and intracellular calcium of G_q/11_ [50].
(iii) G_i_: the role of this class member is to mediate the inhibition of different types of AC. Functional studies have been conducted with PTX, produced by* Bordetella pertussis*. Unlike CTX, PTX decouples the G protein from its receptor and remains inactive and bound to GDP [51].
(iv) G_12/13_: this subunit is expressed ubiquitously in mammals and is composed by two proteins, G_*α*12_ and G_*α*13_ which are also toxin resistant [42]. Meshki et al. reported that the G_12/13_ subunit could regulate changes in cytoskeletal rearrangement when the cell was preparing to migrate. These changes depend on the activation of Rho/Rock which directly modulates the myosin regulatory light chain. Phosphorylation of this protein is associated with the formation of small spherical outgrowths arising from the membrane known as bubbles or blebs, in a process known as blebbing. This process is not always associated with apoptosis but may be associated with the cytoplasmic disorganization at the time of cell migration and Meshki et al.'s study showed how the NK-1 receptor had the ability to interact with the G_12/13_ protein throughout this process [52].
(v) G_o_: this subunit is one of the most abundant G proteins in neuronal and neuroendocrine tissues [53]. Nishimura et al. provided the first evidence of NK-1R's potential to activate G_o_ in Sf9 cells [54]. This subunit signals downstream of frizzled (Fz) GPCRs. G_o_ is crucial for the activation of Wnt-*β*-catenin signaling pathways [42]. While G_o_ is abundant in nervous tissues, its deficiency causes lesions that appear to be mediated mainly by this subunit [42, 55].

The G*βγ* subunit has been less studied than G*α*. The *βγ* complex can be formed by five different *β* subunits and 12 *γ* subunits [42]. At first, it was thought that its role was merely passive but later it was found that it may play a role in the activation of effectors such as PLC*β*, adenylyl cyclases, PI3K, K^+^ ion channels, and Src. All these associations between trimeric G proteins and second messengers lead to a cascade of intracellular events that cause a particular response, depending on cell type.

GPCRs constitute a large family of cell surface receptors which regulate many cellular functions, including cell proliferation, survival and motility, the sense of smell, emesis, and depression. They have recently emerged as key receptors in tumor growth, angiogenesis, and metastasis.

Specifically, interactions involving the G_q/11_ protein occur in several systems and endocrine secretion, vasodilatation, neuromodulation, and activation of monocytes as well as in cell proliferation [56–60]. Therefore, experimental evidence from several recent studies supports the view that alterations in the endocrine system regulated by NK-1R and SP contribute to the development of pathologies such as depression, neural degeneration, alcohol addiction, pain, migraine, inflammatory bowel disease, pruritus, viral infection, bacterial infection, cancer, and emesis [27, 35, 61–65].

#### 1.1.2. Signaling Pathways of NK-1R and SP

**Figure 3:**
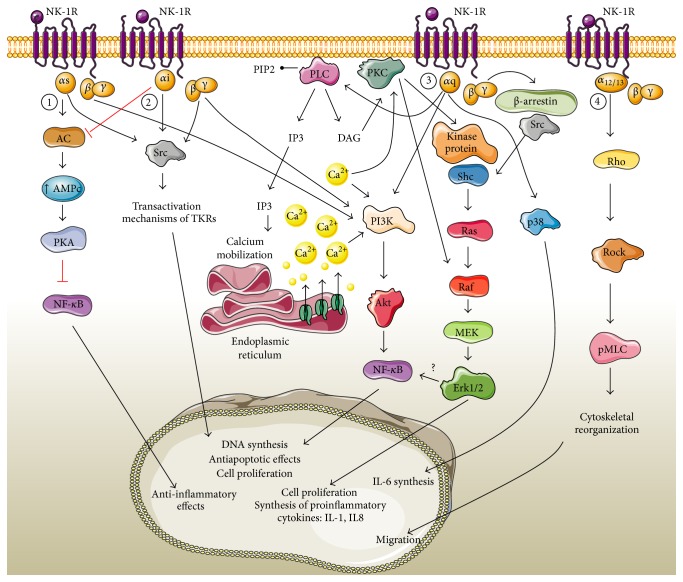
Some of the proposed signaling pathways activated by NK-R. (1) G*α*s activation of AC catalyzes ATP to cyclic AMP (cAMP), which in turn binds to the regulatory subunits of the cAMP-dependent PKA. Usually PKA phosphorylates the CREB transcription factor. CREB binds to the cAMP response element (CRE) of a target gene and negatively affects the activation of NF-кB [49]. (2) Inhibition of the AC is performed by* Pertussis toxin* sensitive Gi protein [48]. Furthermore, Gi and *βγ* subunits enhance Erk1/2 activation after EGFR-mediated transactivation by Src protein [150]. (3) The SP binding to its receptor triggers a GTP-for-GDP exchange on G*α* subunits, thus dissociating G*α*q from G*βγ* and subsequently activating downstream effectors such as PLC. This enzyme catalyzes the conversion of PIP2 in the second messenger IP3 and DAG, stimulating calcium mobilization and PKC activation, respectively [151]. Via nonreceptor protein kinases such as Src or Pyk2, PKC may activate the MAPK pathway but may also activate the Raf protein directly [77]. Another parallel mechanism that regulates MAPK may be developed during NK-1R internalization and its protein recruitment by *β*-arrestins [22, 78]. Although the mechanism is unknown, the Erk1/2 protein is also involved in NF-кB activation [84]. This G*α*q subunit also mediates IL-6 production by activation of p38 MAPK [152]. (4) The G*α*12/13 subunit is responsible for the activation of Rho/Rock which directly regulates the phosphorylation of the myosin light chain (MLC) [52]. Phosphorylation of this protein is associated with cytoskeletal reorganization and cell migration. The *βγ* dimer activates proteins such as Src, PI3K, and PLC [85]. This figure was made using Servier Medical Art collection (http://creativecommons.org/licenses/by/3.0).

The NK-1 receptor signals through different pathways depending on the nature of the G proteins. For example, in glioblastoma cell lines and in many other tumor types, the SP binding causes the accumulation of DAG, which in turn activates PKC. This protein phosphorylates other proteins such as c-Raf-1 and MEK, which phosphorylate tyrosine protein kinase Erk1/Erk2 (also known as p-42/44) of the MAPK protein family [27, 65–69]. The mechanism by which PKC activates ERK is not entirely understood. Discordant results are found in the literature, in which different molecules have been implicated in MAPK activation via GPCRs. These disparities may be explained by differences in the cell culture methods used or the nature of the samples analyzed [70–76]. Subsequently, transcription factors such as c-fos or c-myc are activated and induce DNA synthesis and cell proliferation (Figure 3). Another protein kinase activated by NK-1 receptor is PKC*δ*. Earlier studies by Della Rocca et al. [77] found that PLC activation dependent on both G_q/11_ (*α*1B adrenergic receptor) and G*βγ* subunits (Gi dissociated from *α*2A adrenergic receptor protein) increased cytoplasmic IP3 levels, resulting in an increase in cytoplasmic Ca^2+^. High concentrations of intracellular calcium, probably through calmodulin, lead to kinase activation, called proline-rich tyrosine kinase 2 (Pyk2, English protein tyrosine kinase 2) associated with focal adhesion kinase (FAK). In turn, this Pyk2 activity (now known as PTK2B) regulates kinase protein Src. Src-dependent tyrosine phosphorylation of adaptor proteins such as Shc recruits Grb2-SOS complex to the plasma membrane and initiates the phosphorylation cascade leading the Erk1/2 activation that triggers cell proliferation pathways [77].

According to some studies, MAPK activation depends not only on G proteins and their canonical or classical pathway signaling, but also on the scaffold for the assembly of multiprotein complexes for NK-1R internalization or other GPCRs. In some models such as G*α*q-coupled proteinase-activated receptor 2 (PAR2), the interaction of this receptor with *β*-arrestin internalization proteins causes a retention of Raf-1 and phosphorylated Erk1/2 proteins in the cytoplasm and these proteins cannot be transferred to the nucleus [78].

However, others such as the *β*2-adrenergic receptor (*β*2-AR) are internalized through the complex formed by *β*-arrestin, Src, and Erk [79]. In this case, *β*2-AR receptor activation causes Erk1/2 phosphorylation and induces a different set of cellular responses to those produced by PAR2, since Erk1/2 is not retained in the cytoplasm. These differences may be due to the different scaffolding protein complexes responsible for the distinct subcellular localization of activated kinases for internalization, because they may be responsible for governing the mitogenic potential of each particular signal.

The requirement for *β*-arrestin-dependent endocytosis differs between receptor types. This variation also appears to be cell type-independent, as the two receptors (NK-1R and PAR2) expressed in the same cell line (KNRK) induce the formation of different protein scaffold complexes [22]. Therefore, better studies are needed to identify the GPCR C-terminal end responsible for the internalization process, since this cytoplasmic tail is the key for binding proteins. Feng et al. [23] observed that stimulation of the NK-1 receptor (overexpressed in KNRK cells or naturally expressed in endothelial cells) by SP, activated Erk1/2 via a *β*-arrestin-dependent mechanism. SP induced the formation of a multiprotein complex near the plasma membrane containing *β*-arrestins, Src, and Erk1/2. Once activated, Erk1/2 translocates into the nucleus to induce proliferation and antiapoptotic effects [22].

NK-IR internalization and recycling seems to modulate cellular responses to SP binding, and although SP is degraded, the receptor recovery towards the plasma membrane does not seem to be dependent on new protein synthesis [80].

In addition to its mitogenic activity, SP is also capable of stimulating cytokine release from normal cells and immune cells from the tumor microenvironment in order to promote tumor progression. Moreover, the NF-кB-mediated G protein is involved in several cell types. It has been shown that tachykinins activate NF-кB and stimulate the production of proinflammatory cytokines in several cell types: colon epithelial cells [34], macrophages [81], mast cells [82], T cells [83], and astrocytoma cells [84] and in a lung adenocarcinoma epithelial cell (A549) [56]. However, not all the mechanisms by which this activation occurs are totally known. NF-кB activation by SP is calcium-dependent in astrocytoma cells, but not in colon epithelial cells [34, 81].

Another downstream effector of the various signaling pathways activated by NK-1R is the serine/threonine protein kinase Akt, also known as kinase B (PKB) protein. Phosphoinositol 3-kinase or PI3K is responsible for activating Akt. PI3K can be activated by receptor tyrosine kinases (RTKs) or by integrins transactivation or GPCRs [85]. It is unclear how G proteins activate PI3K, but it is known that PIP2 is converted to PIP3 (capable of activating Akt) by PI3K, whereas PTEN opposes this reaction by dephosphorylating PIP3. The role of G*βγ* subunit in PI3K activation has also been reported, because it is known that there is a direct activation of kinase by the *βγ* dimer [85] (Figure 3). González Moles and colleagues [86] reported that stimulation of the bradykinin receptor (a receptor of the same family as NK-1) by G*α*q and *β*_1_ *γ*_2_ subunits increased Akt phosphorylation due to PI3K and this was responsible for NF-кB activation in HeLa transfected cells. These results suggested that if bradykinin receptor phosphorylation leads to IKK2 activation, then activation of G*α*q, *β*1*γ*2, PI3K and Akt is required (Figure 3). However, these authors reported that inhibition of PI3K and Akt only partially inhibited the activation of downstream proteins, so their study does not exclude other parallel signaling pathways such as those mentioned above, including the MAPK pathway.

Finally, other intracellular signaling mechanisms by which NK-1R is responsible for SP-induced cell shape changes have also been described. These changes depend on the activation of Rho/Rock which directly modulates the myosin regulatory light chain. Meshki and collaborators reported that NK1R has the ability to interact with proteins from the G_12/13_ family [52].

Therefore, all these studies have identified key molecules involved in NK-1R signaling, in various cell types, such as p42/44 protein (MAPK), p38 MAPK, NFкB, PI3K, Akt, Src, EGFR, Rho/Rock, *β*-arrestin, and Pyk2 depicted in Figure 3.

## 2. Distribution of Tachykinin Receptors in the Body

**Figure 4:**
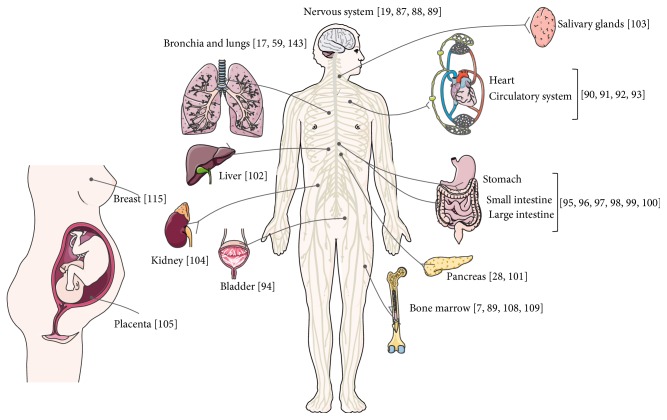
NK-1 receptor distributed in the human body. NK-1R distribution across human tissues. This figure was made using Servier Medical Art collection (http://creativecommons.org/licenses/by/3.0).

As previously mentioned, tachykinins and their receptors are widely expressed in various human systems such as the nervous [19, 87–89], cardiovascular [90–93], genitourinary [94], immune systems, gastrointestinal tract [28, 95–102] and in some tissues such as salivary gland [103], skin, and muscle (Figure 4). Tachykinin receptors are not evenly distributed. The NK-1 and NK-3 receptors are found in the nervous system and in peripheral tissues, whereas the NK-2 receptor is found only in the peripheral tissues (kidney [104], lung, placenta [105] and skeletal muscle) [57, 106, 107]. Specifically, like its higher affinity ligand SP, the NK-1 receptor is involved in cellular responses such as pain transmission, endocrine and paracrine secretion, vasodilation and modulation of cell proliferation. It also acts as a neuromodulator contributing to brain homeostasis but also the sensory neuronal transmission associated with depression, stress, anxiety and emesis. Additionally, the NK-1 receptor is responsible for modulating the immune system's inflammatory response. Expression of the NK-1 receptor has been identified in lymphocytes, monocytes, macrophages, NK cells and microglia. NK-1R is also expressed in bone marrow cells (cells of lymphoid and myeloid lineage) and is considered an hematopoietic regulator [58, 108–112]. Both in normal tissue and during hematopoiesis, NK-1R mediates stimulation effects and NK-2 exerts suppressor functions (when NK-1R is expressed in normal cells, there is a down-regulation of NK-2R) [113, 114].

## 3. NK-1R as a Therapeutic Target

SP, through the NK-1 receptor signal, has been implicated in the regulation of many physiological and pathophysiological functions such as neuronal survival, regulation of cell movement, pain, inflammation, salivation, depression, stress responses, emotions, reward, neurogenesis, vigilance, cancer progression, and emesis [63, 115–123]. Moreover, the tachykinergic system can regulate motility in several cells [52], stimulates platelet aggregation [124], and is present in many human body fluids such as breast milk, blood, saliva, and cerebrospinal fluid [122]. The ubiquity of the SP/NK-1 receptor system in many biological functions and its upregulation under pathological conditions makes this system an important target for several diseases (depression, neural degeneration, alcohol addiction, pain, migraine, inflammatory bowel disease, pruritus, viral infection, bacterial infection, cancer, and emesis [27, 35, 61–65]). Among all these conditions, the NK-1R antagonist has only been subject to clinical development in the treatment of chemotherapy-induced nausea and vomiting (CINV) and in depression. These clinical trials led to the registration of aprepitant by the regulatory agencies EMA and FDA as the first NK-1 receptor antagonist to treat chemotherapy-induced nausea and vomiting.

### 3.1. Emesis

NK-1R and SP are present in brain regions involved in the vomiting reflex (the nucleus tractus solitarius and area postrema) [125]. Aprepitant (MK-869, brand name EMEND) is the first the neurokinin-1 receptor antagonist to be commercialized. When added to a standard regimen of a 5-HT3 receptor antagonist and dexamethasone in cancer patients receiving highly emetogenic chemotherapy, aprepitant improves the complete response (CR) rate in acute CINV. It also improves the CR in delayed CINV when used in combination with dexamethasone compared with dexamethasone alone [126]. The use of aprepitant in patients receiving moderately emetogenic chemotherapy was recently approved after phase III clinical trials had demonstrated its efficacy [127]. Aprepitant is a substrate, a moderate inhibitor, and an inducer of cytochrome P450 (CYP3A4) and CYP2C9. Drug interactions should be monitored when aprepitant is given together with agents affected by CYP3A4 and CYP2C9 isoenzymes.

Aprepitant is the only antagonist with high affinity for the NK-1 receptor approved to date by the US Food and Drug Administration (FDA). It was approved in 2003 for oral administration. In 2008, its prodrug, fosaprepitant, was approved for intravenous use.

These two drugs are the only available agents in this class for preventing chemotherapy-induced and postoperative nausea and vomiting. However, other agents such as netupitant and rolapitant are currently undergoing phase III clinical trials and are expected to be commercialized in the near future [128]. More information on NK-1R as a target for CINV will appear in the following pages of this issue.

### 3.2. Depression

The NK-1R antagonist was tested as a novel antidepressant mechanism in an exploratory phase II clinical trial also using aprepitant [121].

In situations of stress and anxiety, neuropeptides such as SP are released at a rate proportional to the intensity and frequency of stimulation [129]. In fact some studies show that the SP/NK-1R interaction plays an important role in the regulation of emotional behavior [129]. There is evidence that psychosocial help reduces depression, anxiety, and pain and may prolong survival in some cancer patients. Indeed, various forms of stress have been associated with mammary tumorigenesis [130, 131]. Specifically, the NK-1 receptor and SP are involved in emotional responses to stress, suggesting that an alteration in the tachykinergic system may be the key to triggering pathogenesis such as depression (SP expression has been shown to increase during depression [121] whereas the genetic deletion of its receptor induces an anxiolytic and antidepressant effect [132]). It has even been reported that psychotropic drugs modify the expression of genes encoding the synthesis of tachykinin in some areas of the rat brain [133, 134]. Some of these findings suggest that a reduction in SP levels in certain regions of the brain, with NK-1R antagonists, may have a therapeutic effect as antidepressant drug in affective disorders and also in disorders related to cancer. In fact, several publications and reviews have reported experiments correlating emotional behavior (the limbic system) and cancer [35, 135, 136].

### 3.3. Cancer

Experimental evidence obtained in recent years supports the idea that alterations in the neuroendocrine system may contribute significantly to the tumorigenic process. The tachykinins act directly on tumor cells, modulating their responses in terms of proliferation and survival but also contribute indirectly by altering the tumor microenvironment and processes related to tumor progression. SP and its receptor are expressed in a wide variety of tumor cell lines (WERI-Rb-1 and Y-79 from retinoblastoma, U373 MG and GAMG from glioma, SNK-BE(2), Kelly and IMR-32 from neuroblastoma, CAPAN-1 and PA-TU 8902 from pancreatic cancer, Hep-2 from laryngeal cancer, 23132/87 from gastric cancer, and SW-403 from colon cancer) [65, 67, 137] and tumors such as astrocytomas, gliomas, neuroblastomas, pancreatic cancer, melanomas, and breast cancer [28, 86, 123, 135, 138, 139].

It has been estimated that the NK-1R antagonist aprepitant is 45000 times more selective than for the NK-2 receptor and more than 3000 times more selective for the NK-1 receptor than for the NK-3 receptor [140]. This compound has shown antiproliferative properties in tumoral cell lines of glioma, neuroblastoma, retinoblastoma, pancreas, larynx, colon, and gastric carcinoma [62, 64, 141, 142]. A clinical trial for moderate to severe depression, at a dose of 300 mg/day, found this compound to be safe and well tolerated. No statistically significant differences were found comparing adverse events with patients treated with placebo [121]. Although no clinical trials have yet been initiated, there are sufficient preclinical data to believe that NK-1R antagonists may one day be assessed as anticancer agents [3, 5, 28, 35, 62, 64, 122, 123, 137, 138, 141–148].

## 4. Conclusion

The NK-1 receptor is the high affinity receptor of SP, the major mammalial tachykinin. It belongs to the G protein-coupled receptors (GPCRs) family. Tachykinins and their receptors are widely expressed in various human systems. NK-1 receptors are found in the nervous system and in peripheral tissues. Specifically, the NK-1 receptor is involved in cellular responses such as pain transmission, endocrine and paracrine secretion, vasodilation, and modulation of cell proliferation. Also it acts as a neuromodulator contributing to brain homeostasis and sensory neuronal transmission associated with depression, stress, anxiety, and emesis.

NK-1R and SP are present in brain regions involved in the vomiting reflex (nucleus tractus solitarius and in the area postrema). This anatomical localization has led to the successful clinical development of antagonist against NK-1R in the treatment of CINV. Aprepitant is the first NK1R antagonist of this new antiemetic family. Two other NK-1R antagonists have finished clinical trials and it is expected that they will be commercialized in the near future.

## Figures and Tables

**Table 1:** Genes of human tachykinin receptors.

Receptor	Gen	Access number	Chromosomal location
NK-1	TACR1	NM_001058	2p13.1-p12
NK-2	TACR2	NM_001057	10q11-q21
NK-3	TACR3	NM_001059	4q25
